# Controlled Fabrication of Nanoporous Oxide Layers on Zircaloy by Anodization

**DOI:** 10.1186/s11671-015-1086-x

**Published:** 2015-09-29

**Authors:** Yang Jeong Park, Jun Mok Ha, Ghafar Ali, Hyun Jin Kim, Yacine Addad, Sung Oh Cho

**Affiliations:** Department of Nuclear and Quantum Engineering, Korea Advanced Institute of Science and Technology, 373-1 Guseong, Yuseong, Daejeon 305-701 Republic of Korea; Nanomaterials Research Group (NRG), Physics Division (PD), PINSTECH, Islamabad, 45650 Pakistan; Department of Nuclear Engineering, Khalifa University of Science, Technology and Research, Abu Dhabi, 127788 United Arab Emirates

**Keywords:** Zircaloy, Anodization, Oxide layer, Nanopore, Nanotube

## Abstract

We have presented a mechanism to explain why the resulting oxide morphology becomes a porous or a tubular nanostructure when a zircaloy is electrochemically anodized. A porous zirconium oxide nanostructure is always formed at an initial anodization stage, but the degree of interpore dissolution determines whether the final morphology is nanoporous or nanotubular. The interpore dissolution rate can be tuned by changing the anodization parameters such as anodization time and water content in an electrolyte. Consequently, porous or tubular oxide nanostructures can be selectively fabricated on a zircaloy surface by controlling the parameters. Based on this mechanism, zirconium oxide layers with completely nanoporous, completely nanotubular, and intermediate morphologies between a nanoporous and a nanotubular structure were controllably fabricated.

## Background

Electrochemical anodization has been widely used to produce oxide nanostructures on the surfaces of various metals [1–6] and alloys [7–10] due to its simplicity and low cost. The morphology of the metal oxide layer fabricated by anodization is normally nanoporous or nanotubular structure. The main difference between nanoporous and nanotubular structures is the presence of gaps between pores: the pores are interconnected without gaps in nanoporous structures but the pores are splitted due to the gaps in nanotubular structures. Nanotubular metal oxides are useful for the applications to catalysts [11, 12], solar energy conversion [13, 14], hydrophilic surface [15, 16], and sensors [17, 18]. Nanoporous metal oxides are used for corrosion resistance [19, 20], decoration [21], and templates for nanomaterial fabrication [22, 23]. For certain applications, controlled synthesis of the oxide layer to have only nanoporous or only nanotubular structure is crucial. One example is anti-corrosion of a metal using a metal oxide nanostructure [24]. If a nanotubular oxide layer is formed on a metal surface, the layer does not exhibit a good anti-corrosion property. This is because many small gaps exist between the pores and furthermore large cracks are also formed on the nanotubular oxide layers. Water or moisture can then interact with a metal underneath the tubular oxide layer after penetrating into the gaps and cracks, resulting in the corrosion of the metal. However, if a nanoporous oxide layer is created on a metal surface, water or moisture hardly directly meets a metal underneath the oxide layer because no gaps or cracks exist on the nanoporous oxide layer. Consequently, a nanoporous oxide layer can act as a good protective layer for metal corrosion while a nanotubular oxide layer might not show such a good corrosion-resistant behavior.

It has been claimed that the morphologies of the metal oxide nanostructures produced by anodization is dependent on the material: porous morphologies are formed if Al, Ta, and Nb are anodized while tubular structures are formed in the case of Ti, Zr, and Hf when they are anodized in fluoride-containing electrolytes [25]. On the other hand, it has been shown that the resulting morphology of the produced oxide nanostructure is affected by certain anodization parameters [26–28]. So far, a few mechanisms explaining the anodization-induced nanostructure formation and the morphology evolution have been proposed; [22, 26, 28–31] however, the current mechanisms are still controversial.

Here, we propose a mechanism on the morphology evolution of anodic oxide layer to porous or tubular structure and experimentally demonstrate that nanoporous or nanotubular oxide layer can be controllably and selectively fabricated by anodization of zirconium alloys, or zircaloy. The reason behind using zircaloy in the present experiments is to apply this anodization technique to improve the safety of a nuclear power plant under severe accidental conditions. In fact, zircaloy is the most widely used nuclear fuel cladding material in nuclear reactors. When zircaloy comes in contact with high-temperature steam, the zircaloy metal atoms become oxidized from the presence of oxygen molecules in steam. As a result, hydrogen gas is formed from water molecules that have lost the oxygen atoms. If an excessive amount of hydrogen gas is produced, explosion, similar to the Fukushima nuclear power plant accident [32], could occur. However, we suggest that the hydrogen production rate can be dramatically reduced if nanoporous oxide layer is preformed on the zircaloy cladding through anodization. Since zircaloy surface is pre-oxidized through anodization, the oxide layer hinders further oxidation of the zircaloy cladding even though the cladding contacts with steam, thereby preventing hydrogen production due to water splitting. However, for this purpose, the oxide layer prepared on a zircaloy cladding should not have a nanotubular but a nanoporous morphology because nanotubular oxide layer having many gaps and cracks cannot protect the interaction of steam with zircaloy base metal. Therefore, for the application to nuclear reactors, nanoporous oxide structures without gaps and cracks are to be fabricated on the surface of zircaloy cladding.

## Methods

Zircaloy plates (KEPCO Nuclear Fuel Company Ltd., 10 × 40 × 0.7 mm^3^) were used for the anodization experiments. They were cleaned by sonicating in acetone and isopropyl alcohol, followed by rinsing with deionized (DI) water and drying in air. Anodization was carried out using a two-electrode system with a platinum sheet (15 × 40 × 0.5 mm^3^) as a counter electrode and a zircaloy plate as a working electrode. The distance between the two electrodes was 10 mm. Ethylene glycol (95 % purity, Junsei) and glycerol (95 % purity, Junsei) containing ammonium fluoride (NH_4_F, Sigma-Aldrich Corporation, St. Louis, MO, USA) and DI water were used as an electrolyte of the anodization process. All the chemicals and materials were used in their as-received forms without any further purification. A direct current power supply with a maximum capacity of 1000 V and 1 Å was used for the electrochemical treatment. The anodization experiments were performed in a dry glove box at room temperature. After the experiments, the samples were rinsed with DI water and subsequently dried in air.

The structural morphologies of the anodized samples were examined by a field emission scanning electron microscope (FESEM, Nova230, FEI, USA). For the measurement, the anodized oxide layer was mechanically cracked on purpose. The chemical composition of the sample was characterized using energy dispersive X-ray spectroscopy (EDS) (EDAX Genesis attached to the FESEM). The crystalline structure was identified with the help of glancing angle X-ray diffractometer (GAXRD, D/MAX 2500 V, Rigaku Corporation, Tokyo, Japan) with Cu Kα radiation (*k* = 1.5406 Å).

## Results and Discussion

If a metal is electrochemically anodized in an electrolyte, several reactions occur between the metal and the electrolyte. In particular, the following reactions can occur in our experiments where zircaloy is anodized in an aqueous fluoride-containing electrolyte.

1 $$ {\mathrm{Zr}}^{4+}+2{\mathrm{O}}^{2-}\to {\mathrm{Zr}\mathrm{O}}_2, $$

2 $$ {\mathrm{ZrO}}_2+6{\mathrm{F}}^{-}+4{\mathrm{H}}^{+}\to {\left[{\mathrm{ZrF}}_6\right]}^{2-}+2{\mathrm{H}}_2\mathrm{O}, $$

3 $$ {\mathrm{Zr}}^{4+}+6{\mathrm{F}}^{-}\to {\left[{\mathrm{Zr}\mathrm{F}}_6\right]}^{2-}\cdot $$

In the reaction (1), oxygen ions that are produced from water combine with a metal cation to form a metal oxide. In the reaction (2), fluorine ions that are produced from an electrolyte react with the preformed metal oxide, forming soluble compounds. As a result, the preformed metal oxide is etched and nanometer-sized pores are created on the metal oxide. These two reactions are enhanced by an electric field that is generated by an anodization voltage because anions are involved in the reactions. Etching is further accelerated through the reaction (3), where a metal cation is directly ejected to the electrolyte due to the electric field. The oxidation (1) and etching (2, 3) reactions compete with one another during the anodization process, and an oxide layer with nanometer-sized pores is formed.

In addition, like in Eq. (1), a fluorine ion can combine with a metal cation to form a metal fluoride through the following reaction:

4 $$ {\mathrm{Zr}}^{4+}+4{\mathrm{F}}^{-}\to {\mathrm{Zr}\mathrm{F}}_4\ . $$

Both fluorine ions and oxygen ions in an electrolyte can migrate into a metal substrate due to the electric field, generating metal oxide through reaction (1) and metal fluoride through reaction (4). Hence, a barrier layer that consists of metal oxide and metal fluoride is generated between the pores and a metal substrate. Since a fluorine ion is less bulky than an oxygen ion, fluorine ions migrate inward more rapidly and deeply than oxygen ions [28, 31]. As a result, an oxide-rich sublayer and a fluoride-rich sublayer are generated in the top and bottom of the barrier layer, respectively. These two oxide and fluoride materials are displaced towards the pore walls by a flow mechanism [22, 33], forming a double layer between two neighboring pores: an oxide-rich layer and a fluoride-rich layer in sequence from the pore center [34]. The fluoride compounds in the interpore regions are chemically dissolved by an aqueous fluoride-containing electrolyte as follows [34–36]:

5 $$ {\mathrm{ZrF}}_4+{\mathrm{F}}^{-}+2{\mathrm{H}}_2\mathrm{O}\to {{\mathrm{OH}}_3}^{+}{{\mathrm{ZrF}}_5}^{-}+{\mathrm{OH}}^{-} $$

Due to the reactions (1)–(5), self-organized metal oxide nanostructures are fabricated if a metal is anodized. The morphologies of the resulting oxide layers are normally nanotubes or nanopores. Here, we propose that porous or tubular nanostructures can be controllably fabricated by tuning the reaction rates. The reactions (2) and (3) result in pores on a metal substrate because a metal substrate and a metal oxide are etched away, and we call these reactions as pore etching reactions. The rate of pore formation is determined by the pore etching rate, which is proportional to the anodization current [37]. A high anodization current reflects a high pore etching rate and a fast pore formation. The magnitude of the anodization current generally decreases with time [25], suggesting that pores are rapidly formed during an initial anodization stage but grow slowly as anodization proceeds. The pore formation rate can be increased by increasing the anodization voltage because the pore etching reactions are strongly affected by the applied electric field. The average length of the created pore is determined by the total charge flowing through the anode [7], which is calculated by integrating the anodization current with time. In contrast, reaction (5) leads to the change in the morphology of the oxide layer from nanopores to nanotubes because the interpore regions are chemically dissolved. We call the reaction (5) as interpore dissolution reaction. Reaction (5) suggests that the interpore dissolution rate is influenced by water and fluoride contents in an electrolyte. We confirmed that nanoporous morphologies were not changed when the anodized samples were placed in F^−^ free electrolytes such as in NH_4_Cl and NH_4_NO_3_ solutions, which demonstrates that the presence of F^−^ ions is crucial for the transformation in the aging process. Therefore, the pore etching rate or the interpore dissolution rate can be independently or selectively adjusted by the anodization parameters, and then, the morphology of the resulting metal oxide can be controlled to be a porous or a tubular nanostructure.

**Fig. 1:**
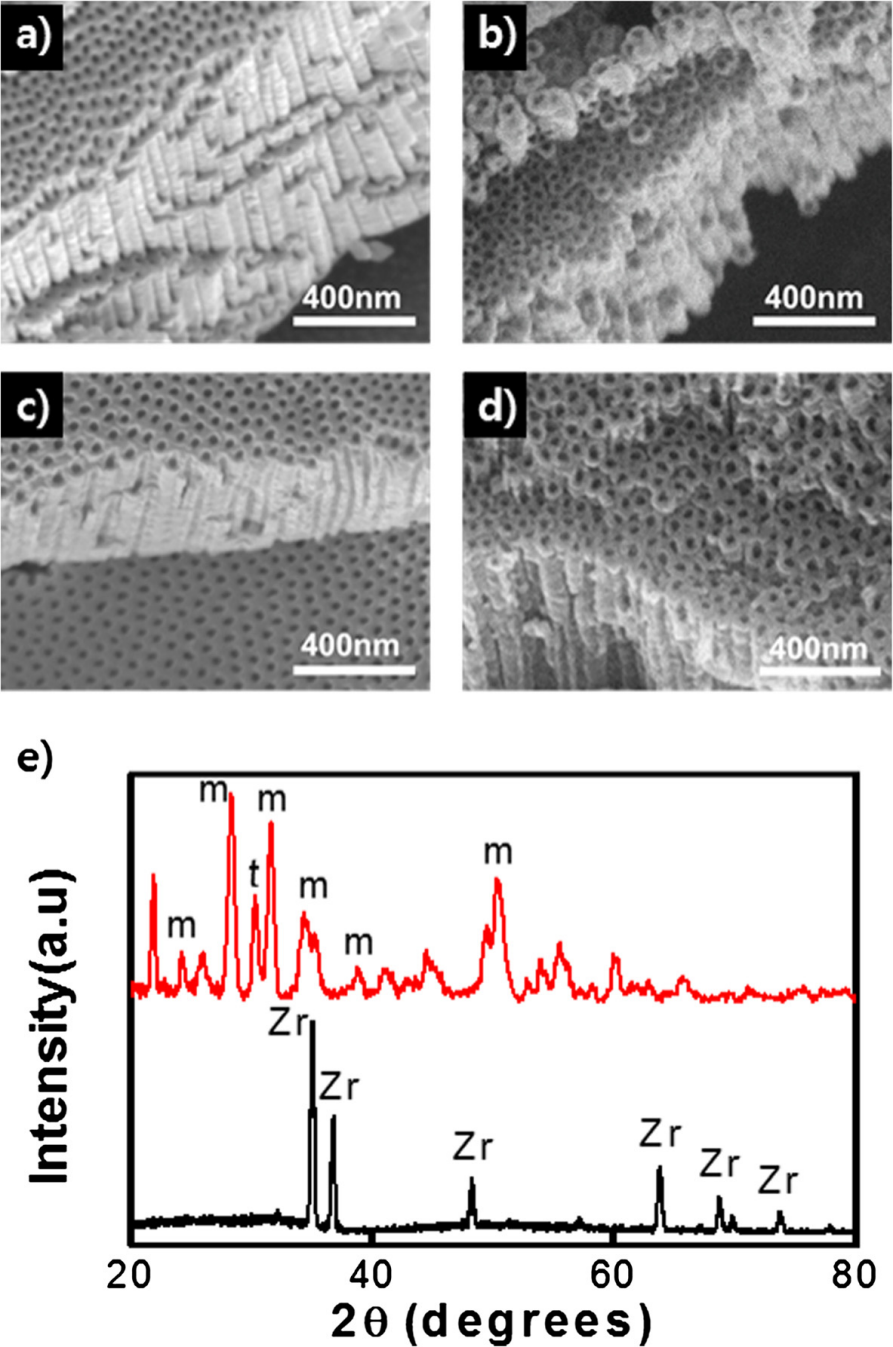
FESEM images of close-packed ZrO_2_ nanoporous structures prepared in ethylene glycol with 1 wt% H_2_O and 0.3 wt% NH_4_F for 5 min at different voltages of **a** 30 and **c** 90 V, respectively. **b** and **d** are nanotubular structures derived from **a** and **c** by aging for **b** 40 min and **d** 2 h, respectively. **e** XRD patterns of an as-anodized (*black line*) and an annealed (*red line*) ZrO_2_ nanoporous structure. The annealing process was carried out at 500 °C for 12 h in a vacuum (*red line*). *m* and *t* denote monoclinic and tetragonal phases of crystalline ZrO_2_

This fact was demonstrated in our experiments (see details in “Methods”). At first, regularly ordered porous ZrO_2_ nanostructures were fabricated by adjusting the anodization parameters. Figure 1a, c shows that hexagonally close-packed nanoporous structures were formed when a zircaloy plate was anodized in ethylene glycol with 1 wt% H_2_O and 0.3 wt% NH_4_F for 5 min at different voltages, 30 and 90 V, respectively. The average diameters of the pores produced at 30 and 90 V are 7 and 25 nm, respectively. The thickness of the oxide layer was increased from 3 to 8 mm when the anodization voltage was increased from 30 to 90 V. Both the cross-sectional view and the top view images clearly display that the surfaces have well-ordered nanoporous morphologies. No gaps or cracks exist between the pores. XRD spectrum reveals that the oxide layer formed by the anodization is amorphous: only Zr diffraction peaks were observed in the spectrum (black line in Fig. 1e). However, when the sample was annealed at 500 °C for 12 h in a vacuum, the amorphous oxide layer became monoclinic-phase crystalline ZrO2 with minor tetragonal phase (red line in Fig. 1e).

**Fig. 2:**
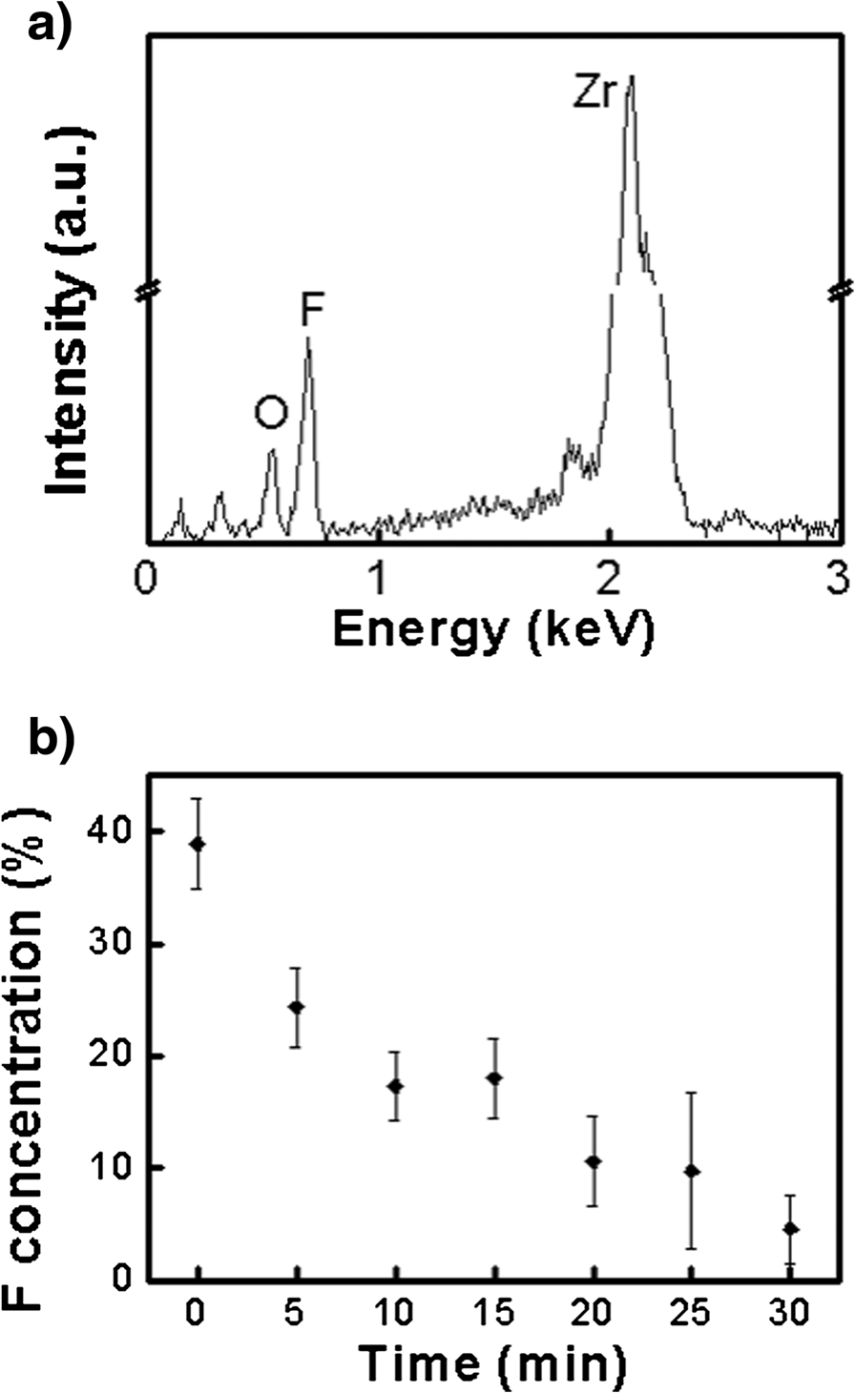
**a** EDS spectrum of an as-anodized ZrO_2_ nanoporous structure. **b** The atomic concentration of F in the ZrO_2_ nanostructure as a funtion of aging time

The porous structure of the oxide layer can be simply transformed into a tubular structure by an aging process [38], which is the immersion of an as-anodized specimen in an electrolyte without applying voltage. An aging process can start following an anodization process in the same electrolyte only by switching off a power supply. Since no voltage is applied during the aging process, the pore etching reactions can hardly occur. However, the interpore dissolution reaction transpires if water and fluoride exist in an electrolyte. As a result, the interpore regions are gradually dissolved while almost no change happens inside the pores during the aging process. This was confirmed in our experiments. The porous ZrO_2_ nanostructures shown in Fig. 1a, c were aged for 40 min and 2 h in the electrolyte, respectively, after anodization. As shown in Fig. 1b, d, the porous structures were completely converted into tubular structures. Figure 2a shows the EDS spectra of the oxide nanostructures. The presence of F as well as Zr and O suggests that interpore regions or pore walls contain fluoride compounds. Comparatively high atomic concentration of F in the as-anodized nanoporous oxide layer reflects that a large amount fluoride compounds like ZrF_4_ are present in the pore walls or in the interpore regions. However, the atomic concentration of F decreases as the aging time is increased (Fig. 2b). This reveals that more and more fluoride compounds in the interpore regions are dissolved with aging time. The results shown in Fig. 1 indicate that although a porous oxide nanostructure is produced by anodization, it can be transformed into a tubular structure if the anodized sample is not extracted from the electrolyte for a certain time after the anodization.

**Fig. 3:**
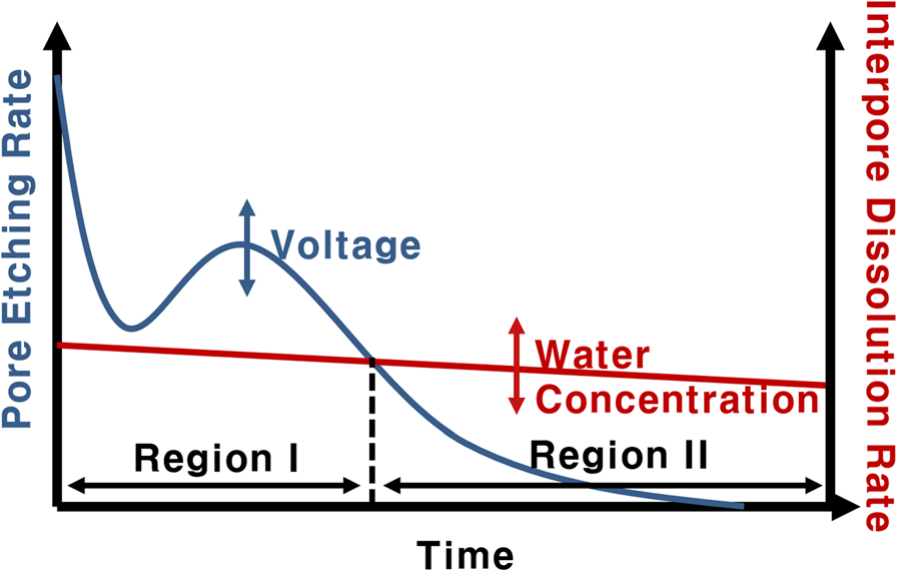
Schematic diagram showing the pore etching rate and the interpore dissolution rate as a function of time

**Fig. 4:**
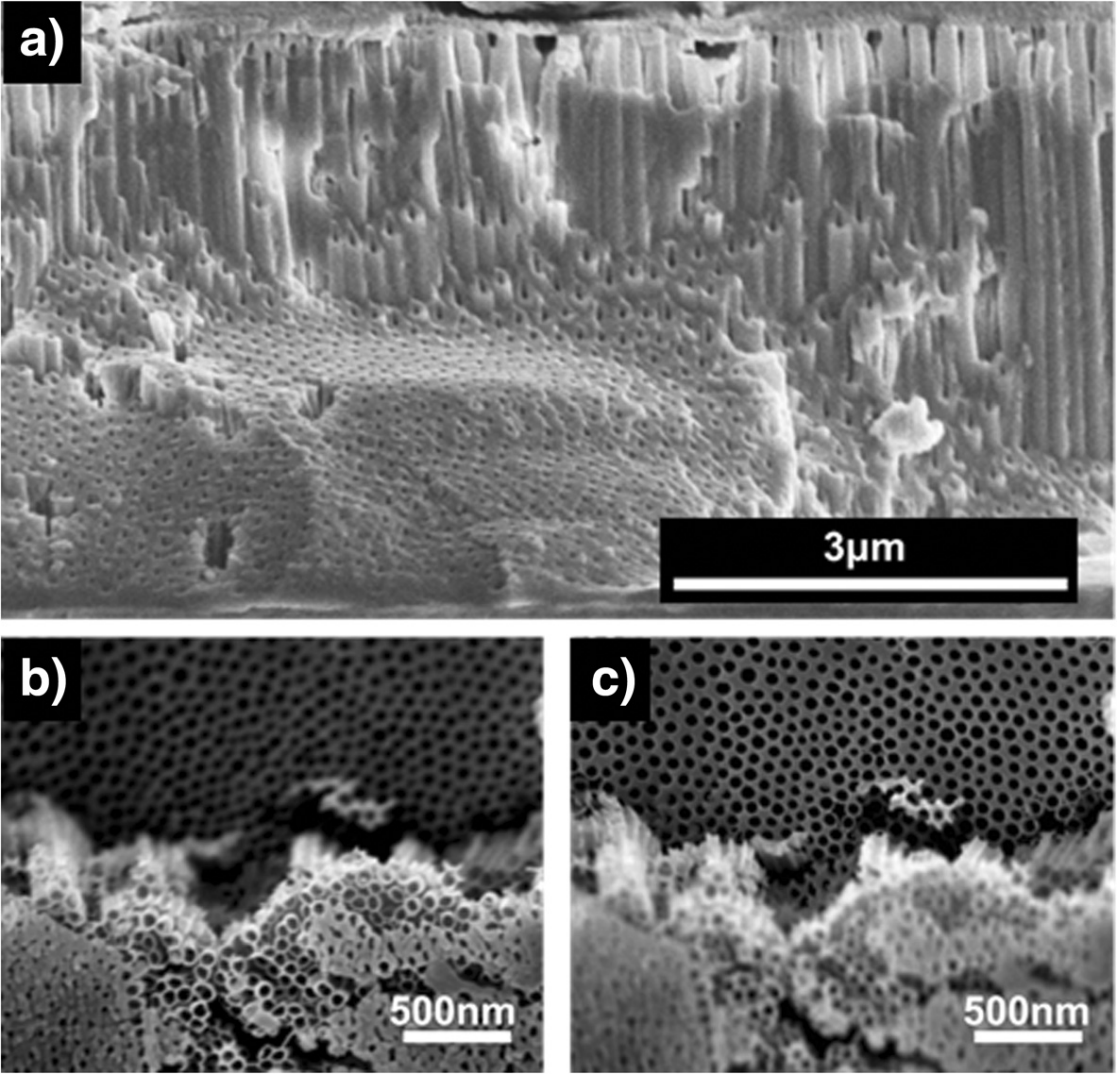
FESEM images showing an intermediate process that a ZrO_2_ porous morphology is transformed into a tubular morphology: **a** cross-sectional, **b** *top*, and **c** *bottom* images of the oxide layer

**Fig. 5:**
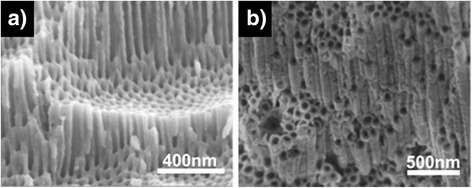
FESEM images of **a** a nanoporous and **b** a nanotubular ZrO_2_ layers fabricated at different anodization time of **a** 5 min and **b** 2 h, respectively. Both oxide layers were prepared by anodization of zircaloy in ethylene glycol containing 0.05 wt% H_2_O and 0.3 wt% NH_4_F at 90 V

Morphology control of the oxide nanostructures can also be achieved by changing the anodization time. During an anodization process, not only the pore etching reactions but also the interpore dissolution reaction occurs. As described earlier in the temporal behavior of the anodization current, pores start to grow very rapidly during an initial anodization stage. At the same time, interpore materials are also removed due to the interpore dissolution reaction, converting the preformed porous structure to a tubular structure from the top layer. During an initial anodization stage (region I in Fig. 3), the pore etching rate is higher than the interpore dissolution rate. As a result, if anodization is carried out for a short time, the produced oxide has a nanoporous morphology since the amount of dissolved interpore materials is negligible. However, the pore etching rate is drastically decreased compared to the initial value after a certain anodization time. In contrast, the interpore dissolution rate is slowly decreased with time as water content in an electrolyte is gradually decreased during anodization. This suggests that the interpore dissolution rate can be higher than the pore etching rate after some time. Therefore, if anodization is carried out for a long time, a porous oxide layer that is initially formed is gradually converted into a tubular structure from the top layer with time. As a consequence, the oxide layer has an intermediate morphology between nanoporous and nanotubular structures, or the oxide layer consists of nanotubes in the top region and nanopores in the bottom region. This was experimentally confirmed. Zircaloy was anodized in glycerol with 0.05 wt% H_2_O and 0.3 wt% NH_4_F at 200 V for 60 min. Note that glycerol has higher viscosity than ethylene glycol, and accordingly, the anodization reactions in glycerol occur much more slowly than those in ethylene glycol [39]. This slow reaction allows to readily observe an intermediate process that a porous morphology is transformed into a tubular morphology. As clearly shown in Fig. 4, the fabricated oxide has an intermediate morphology: the bottom oxide layer has a porous morphology but the top layer has a tubular morphology. The length of the top nanotubes is determined by the length that the interpore region is dissolved, and this transformed length is calculated by the multiplication of the interpore dissolution rate and the anodization time. Thus, a completely tubular oxide nanostructure can be created if the anodization time is long enough. Figure 5 confirms this fact. When zircaloy was anodized for 5 min, the resulting oxide layer was nanoporous (Fig. 5a). However, when the zircaloy was anodized for 2 h, completely nanotubular oxide layer was produced (Fig. 5b). All the other anodization parameters except the anodization time were the same for both experiments: anodization was carried out at 90 V in ethylene glycol containing 0.05 wt% H_2_O and 0.3 wt% NH_4_F. Note that the thickness of the oxide layer was increased from 8 to 15 mm when the anodization time was increased from 5 min to 2 h. Consequently, porous or tubular oxide nanostructures can be controllably fabricated only by changing the anodization time.

**Fig. 6:**
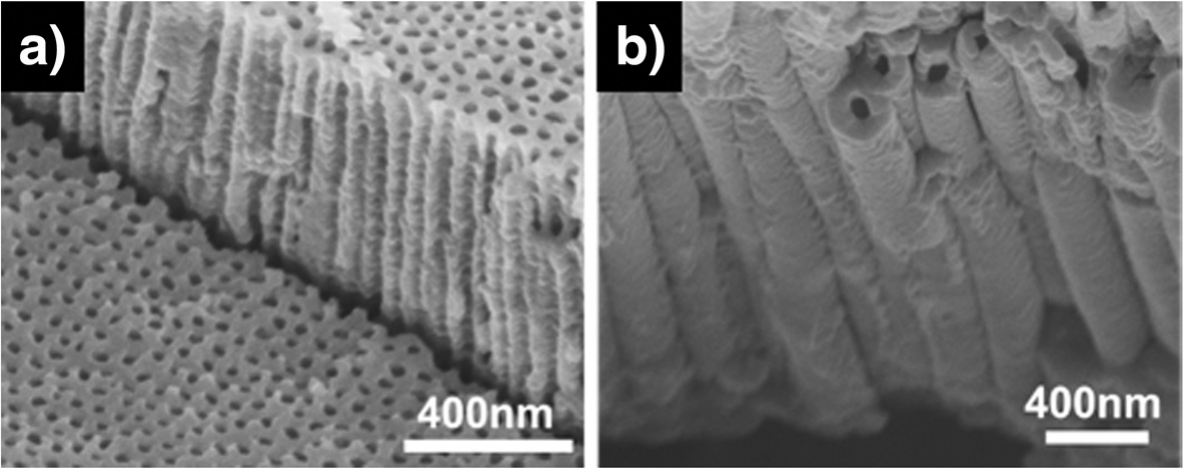
**a** FESEM image of **a** a nanoporous and **b** a nanotubular ZrO_2_ layers fabricated at different water content of **a** 0.5 and **b** 10 wt% in an electrolyte, respectively. Both oxide layers were prepared by anodization of zircaloy in ethylene glycol containing 0.3 wt% NH_4_F at 90 V for 10 min

Moreover, the water content in an electrolyte also affects the resulting morphology of the anodized oxide layer. As can be seen in Eq. (5) and Fig. 3, the interpore dissolution rate is increased by increasing the water content. If water content in an electrolyte is high enough, the dissolution length of the interpore regions becomes large even at a short anodization time. Consequently, nanoporous structures are produced at low water content but nanotubular structures are readily formed at high water content. Figure 6 verifies this fact. When a zircaloy plate was anodized in ethylene glycol containing 0.5 wt% H_2_O and 0.3 wt% NH_4_F at 90 V for 10 min, nanoporous oxide layer was produced. However, when the water content was increased from 0.5 to 10 wt% while keeping all other parameters the same, the anodized oxide layer became completely nanotubular structure. Note that the water content also affects the pore size in addition to the porous/tubular morphology. The pore size was increased from 40 to 70 nm when the water content was increased from 0.5 to 10 wt% in Fig. 6.

It should be noted that anodization voltage is not a critical parameter to determine that the morphology of the resulting oxide layer is nanopores or nanotubes. Higher anodization voltage leads to higher anodization current. As a consequence, pore etching rate and correspondingly the length of pores is increased. However, whether the final morphology of the oxide layer is nanoporous or nanotubular is determined mainly by how much the interpore regions are dissolved. Since the interpore dissolution reaction (5) is a chemical reaction that is not affected by an electric field, anodization voltage does not affect the porous or tubular morphology. As demonstrated previously, if anodization time is sufficiently small or if water content is sufficiently low, the dissolution of the interpore regions can be negligible, and thus, the resulting oxide layer has a completely nanoporous structure (Figs. 1a, c, 4a, and 5a). However, if the product of anodization time and water content is not so small and not so large, an intermediate structure comprising a top nanotube layer and a bottom nanopore layer is created (Fig. 4). If the product of anodization time and water content is large enough, a completely nanotubular oxide layer is fabricated (Figs. 1b, d, 4b, and 5b).

## Conclusions

In conclusion, we have proposed a model to explain why the morphologies of anodized metal oxide layers are evolved to nanoporous or nanotubular structures. Nanoporous structures are initially formed by anodization, but the degree of interpore dissolution determines that the final morphology of the oxide layer is nanoporous or nanotubular. The degree of the interpore dissolution can be tuned by changing the anodization parameters such as anodization time and water content in an electrolyte as well as aging time. Consequently, nanoporous or nanotubular oxide morphology can be selectively fabricated on a metal surface by controlling the parameters. This model was demonstrated through the anodization experiments of zircaloy. We suggest that the proposed model can also be applied to other metals and alloys for the preparation of morphology-controlled oxide nanostructures on the surface. Therefore, the anodic metal oxides can exhibit enhanced performances for various applications
